# One Medical College for Louisville1Louisville Monthly Journal of Medicine and Surgery, September, 1908.

**Published:** 1908-10

**Authors:** 


### One Medical College for Louisville.[1]

THE final details of the merger of the medical colleges of Louisville have been worked out by the committee composed of Drs. J. M. Bodine, Chairman; C. W. Kelly, W. H. Wathen, T. C. Evans, L. S. McMurtry, J. B. Marvin, H. H. Grant, M. F. Coomes, H. B. Ritter and R. B. Gilbert.

By the terms of agreement the property of the Louisville and Hospital Medical College, located at First and Chestnut streets, and the Kentucky School of Medicine, on Sixth street between Chestnut and Walnut, is acquired. The City Council will be asked by the trustees of the University to appropriate $30,000 for the equipment of the laboratories of the medical department.

The following officers were elected. Dr. J. M. Bodine, president; Dr. J. B. Marvin, vice president; Dr. T. C. Evans, dean, and Dr. P. F. Barbour, secretary. The Executive Committee composed of: Dr. W. H. Wathen, Dr. C. W. Kelly, Dr. L. S. McMurtry, Dr. H. H. Grant, the president and dean ex-officio members of the Executive Committee. The AuditingCommittee is composed of Drs. H. E. Tuley, M. F. Coomes, H. B. Ritter. Purveyor, Dr. H. N. Leavell. Committee to take inventory of the laboratory of the three schools are: Drs. J. B. Marvin, E. S. Allen, B. J. O'Connor and H. A. Davidson.

The following major faculty was elected by the Commission: Drs. William Bailey, J. M. Bodine, Thomas C. Evans, William E. Grant, Henry A. Cottell, L. L. Solomon, Virgil E. Simpson, Henry M. Goodman, Edw. R. Palmer, Joseph B. Marvin, John G. Cecil, Richard B. Gilbert, Samuel E. Woody, William O. Roberts, Thomas L. Butler, J. Garland Sherrill, Argus D. Willmoth, Turner Anderson, Louis Frank, William B. Doherty, Henry Enos Tuley, J. Morrison Ray, Adolph O. Pfingst, Isadore N. Bloom, Charles W. Hibbitt, Bernard Asman, Isaac Lederman, Carl Weidner, Lewis S. McMurtry, Frank C. Wilson, Samuel G. Dabney, John Edwin Hays, H. Horace Grant, Philip F. Barbour, Edward Speidel, George A. Hendon, Walter F. Boggess, Ezra O. Witherspoon, H. A. Davidson, William H. Wathen, Martin F. Coomes, Henry Orendorf, Fouche W. Samuel, William A. Jenkins, John R. Wathen, George B. Jenkins, Arthur J. Boyd, Oscar C. Dilly, Ellis S. Allen, Curran Pope, Granville S. Hanes, Gay-

1. Louisville Monthly Journal of Medicine and Surgery, September, 1908.

lord Hall, C. W. Kelly, Irvin Abell, B. F. Zimmerman, H. N. Leavell, S. J. Meyers, B. J. O'Connor, Ellis Duncan and W. C. Dugan.

---

THE tuberculosis congress at Washington as was expected did little but get formally under way at the first official session held in the National Museum, September 28, 1908. Great results are expected from the conference ultimately and expectations will probably be in large measure realised. One important outcome of the meetings, says the *Buffalo Express,* will no doubt be dissemination of confidence that tuberculosis is not necessarily fatal and of the best ways in which to fight the disease. Confidence in the possibility of cure and realisation that the best methods of combating the plague individually are practically within everybody's reach should be of immense service to the world.

---

NEWS comes through the press despatches that a disease of the upper air passages, due to the forest fires, is prevalent in various parts of the country, particularly in New England. The patients have an itching in the throat which awakens them from sleep. This is followed by severe coughing spells, which ordinary throat remedies fail to stop. The entire throat structure is depressed and irritated similar to the discomfort experienced in tonsilitis.

It is expected, of course, that the ailment will disappear when rain puts out the fires and clears the atmosphere. Happily, the long desired relief has come at last, for as we write rain is falling in torrents, the atmosphere is cleared, and vegetation is reviving.

---

TARRANT'S Effervescent Seltzer Aperient is one of the most efficient and agreeable alkaline laxatives that can be administered in the therapy of rheumatic and gouty affections. It is a scientific preparation entirely within the bounds of ethical propriety, against which no taint of deception or fraud has ever been charged, and no suspicion has ever arisen. It is available in many kidney disorders and when given under the advice of a physician gives satisfactory results; so also in many other affections when appropriately administered.

---

FOR the first time in several years in this city the degree of Master of Pharmacy was conferred upon five distinguished men from different sections of the United States, who have attained distinction in the art of preparing medicines and drugs. Those who received this honor were Samuel W. Fairchild, of New York; Horatio Nelson Fraser, of New York; John F. Hancock, of Baltimore; S. A. D. Sheppard, of Boston, and William McIntyre, of Philadelphia.—*Evening Bulletin, Philadelphia.*

